# Classification of Whole-Body Postural Discomfort Using Cluster Analysis

**DOI:** 10.3390/ijerph17228314

**Published:** 2020-11-10

**Authors:** Jaejin Hwang, Kyung-Sun Lee

**Affiliations:** 1 Department of Industrial and Systems Engineering, Northern Illinois University, DeKalb, IL 60115, USA; jhwang3@niu.edu; 2 Department of Industrial Health, Catholic University of Pusan, Busan 46241, Korea

**Keywords:** whole-body posture, discomfort, cluster analysis, joint angle, classification

## Abstract

The objectives of this study were to evaluate the effect of gender and postures of the neck, trunk, and knee on overall postural discomfort, and to classify combined postures into different postural discomfort groups. A total of 95 participants (42 males and 53 females) performed 45 different static postures, which were a combination of 3 neck angles, 5 trunk angles, and 3 knee angles, and rated the perceived postural discomfort. Non-hierarchical K-means cluster analysis was employed to classify the 45 different combined postures into several postural discomfort groups. Postural discomfort was significantly affected by gender and postures of the neck, trunk, and knee (*p* < 0.001). Three clusters (high, medium, and low discomfort) were identified and the postural discomfort was significantly different between clusters (*p* < 0.001). The high discomfort group consisted of mostly males with high knee and trunk flexion angles and a moderate neck flexion angle. The low discomfort group was female-dominant with low neck and trunk flexion angles and a moderate knee flexion angle. The different flexibility (stiffness) of the joint motions between genders may affect the gender difference in postural discomfort. The knee and trunk postures were critical to the postural balance, which may affect the perception of whole-body discomfort. This result will be useful for developing and improving postural observation tools.

## 1. Introduction

Awkward postures have been one of the primary components that are associated with work-related musculoskeletal disorders (WMSDs). Previous studies have shown that awkward postures were associated with the increased discomfort and strain of workers [1,2,3,4]. There have been several postural observation tools used to assess postural stress. Typical postural assessment tools include rapid upper limb assessment (RULA) [5], rapid entire body assessment (REBA) [6], and the Ovako working-posture analyzing system (OWAS) [7]. The benefit of these tools is that they do not need specific equipment and can be practically used in the field. These techniques have a similar approach to calculating the risk scores of body segments and determine the action level. One drawback is that coordination between body segments is not thoroughly considered when computing the final score [3]. This could under- or overestimate the demand from whole-body postures.

Postural discomfort or a psychophysical approach has been used to identify the perceived load of various postures. It is known that a feeling of discomfort is determined by sensor and information processing, which are associated with the state of the participant [8]. The discomfort is also interpreted as a clustered descriptor of fatigue, restlessness, pain, biomechanics, strain, and circulation [9]. Musculoskeletal discomfort could be a precursor of musculoskeletal pain, and it can be considered as an important measure to prevent severe pain or injuries [10].

Postural discomfort measurement is considered to be a useful methodology since it does not require biosensors or equipment. A perceived discomfort-based ranking system has been developed to assess the stress of non-neutral static postures [11]. Previous studies showed that increasing the upper arm elevation and abduction were related to an increasing trend of discomfort [12,13,14]. It was also found that there was an interaction effect from the back and shoulder postures on discomfort ratings [3].

Cluster analysis has been broadly used to identify patterns of associations and classify data into several homogeneous groups. This technique has been applied to classify different postural and movement characteristics regarding ergonomics. A previous study used K-means cluster analysis to classify 18 full-swing motions into 2 or 3 different ranges of motion that consisted of similar joint kinematics [15]. Driver’s postural adoptions for different vehicle types have also been identified via cluster analysis using joint angle data [16]. Major factors affecting comfort and discomfort in sitting have also been identified using cluster analysis [9].

Existing postural observation tools have evaluated individual body parts separately and determined the total stress level by simply combining or summating each segment’s results. However, whole-body discomfort could be more affected by improper posture occurring in certain body parts. There has been a lack of studies investigating how different body segment angles were associated with the overall postural discomfort levels of a variety of combined (or coordinated) postures. The objectives of this study were (1) to evaluate the effect of gender and postures of the neck, trunk, and knee on postural discomfort, and (2) to classify the combined postures into different postural discomfort groups. It was hypothesized that postural discomfort would be significantly affected by the gender and postures of the neck, trunk, and knee.

## 2. Materials and Methods

### 2.1. Participants

A total of 95 young adult university students (42 males and 53 females) participated in this study. Participants were randomly selected from the university. For the inclusion criteria, participants needed to not have a current physical symptom or previous history of MSDs over the past 2 years. The mean and standard deviation (SD) of the age, height, and body weight of the participants were 22.5 ± 2.8 years, 170.8 ± 6.5 cm, and 62.1 ± 8.1 kg, respectively (Table 1). The experimental protocol was approved by the Institutional Review Board (CUPIRB-2019-064). Participants gave their informed consent prior to the experimental session.

### 2.2. Experimental Protocol

**Figure 1:**
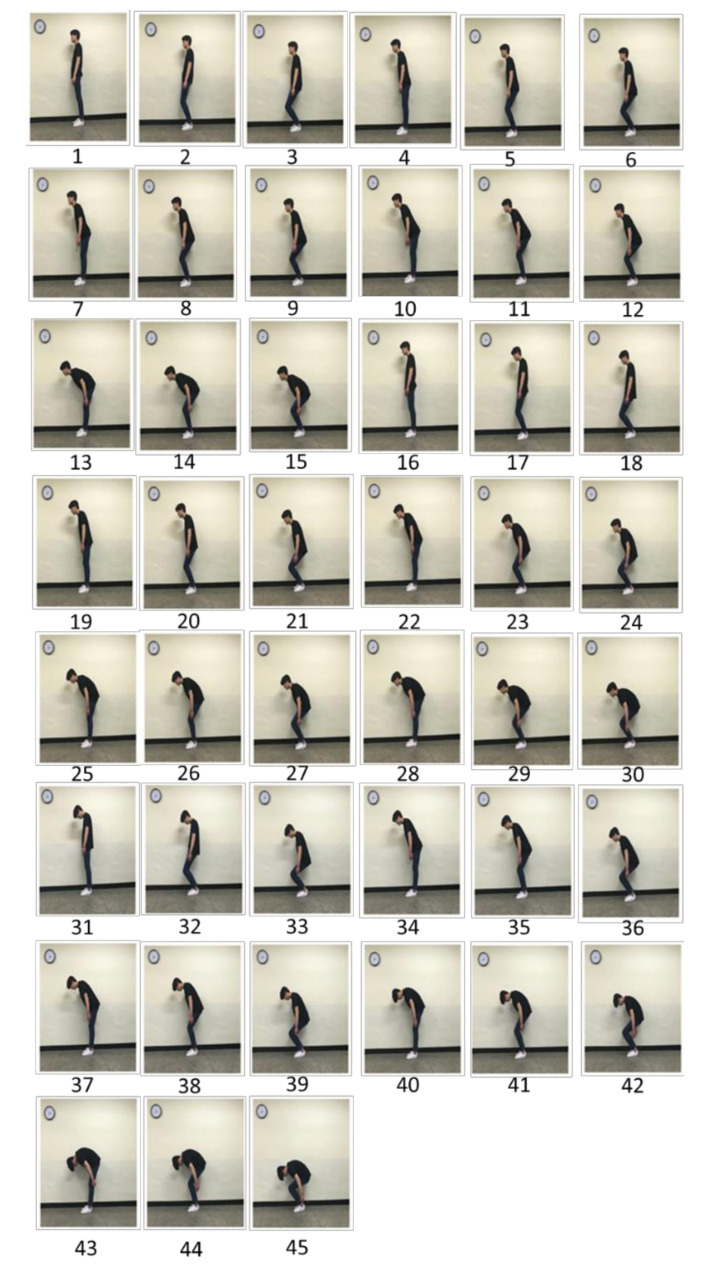
Examples of the 45 combined postures in terms of the neck flexion (0°, 20°, and 40°), trunk flexion (0°, 20°, 40°, 60°, and 80°), and knee flexion (0°, 30°, and 60°) angles. The posture codes match with the description in Table 2.

The experiment was conducted at the laboratory, where the temperature and relative humidity were controlled to be around 20 °C and 60%, respectively. Each participant performed 45 different combined postures, which were a combination of 3 neck flexion angles (0°, 20°, and 40°), 5 trunk flexion angles (0°, 20°, 40°, 60°, and 80°), and 3 knee flexion angles (0°, 30°, and 60°) in the sagittal plane. The levels of the body segment angles and their measurement procedures were based on the REBA checklist [6]. The order of the combined postures was fully randomized. For each combined posture, the operator utilized a goniometer to set up the exact posture. Participants maintained the assigned posture for 3 s. The time was controlled using a beep sound. After performing each posture, a 1 min break was given to reduce fatigue. After performing each posture, the participant rated their overall postural discomfort using the 10-point Likert scale (1: least discomfort to 10: greatest discomfort). Figure 1 shows examples of the combined postures and Table 2 shows the descriptions of the combined postures.

### 2.3. Data Analysis

The independent variables were gender, neck flexion angle, trunk flexion angle, knee flexion angle, and the combined posture. The dependent variable was postural discomfort. The normality of the data was initially diagnosed. The data met non-parametric assumptions; therefore, a Kruskal–Wallis test was conducted to assess the effects of gender, the joint angles, and the combined posture on the postural discomfort. For the significant variables, a series of post-hoc Wilcoxon signed-rank tests identified where differences existed between the postural groups. Statistical significance was set as *p* < 0.05, and the means and standard errors of variables were summarized.

Non-hierarchical K-means cluster analysis was employed to classify the 45 different combined postures into several postural discomfort groups using Minitab 18 (Minitab Inc., State College, PA, USA). For the similarity measure, the Euclidean distance was computed between each data point and centroid of its cluster. The input variables were gender and the joint angles of the neck, trunk, and knee. Gender was chosen because it has been known to influence joint motion stressfulness and discomfort [12,17]. Joint angles were considered as the inputs because previous studies found that individual joint angles were associated with whole-body discomfort [18,19]. Standardization of the variables was conducted to compensate for different scales among variables and provide equal weights to the variables.

The initial k = 2, 3, and 4 were empirically tested to find the optimal number of clusters in this study. The criteria for determining the optimal number of clusters was (1) visual examination of the mean and proportional data in clusters should not be complicated, (2) the number of data points in each cluster should not be too small or too large to prevent excessive within-group variability, and (3) the resulting postural discomfort values should be distinguishable between clusters [15]. The Kruskal–Wallis test was additionally conducted to analyze the statistical differences in postural discomfort between clusters at a significance level of 0.05.

## 3. Results

**Figure 2:**
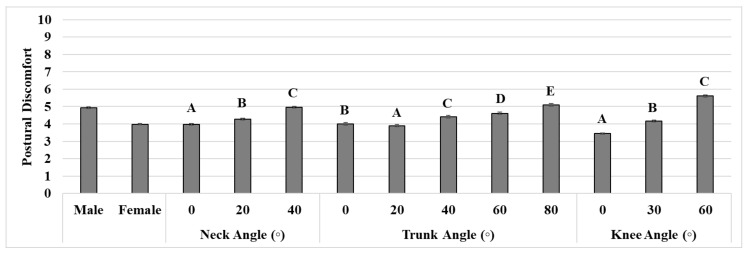
Means and standard errors (error bars) of postural discomfort in terms of the gender, neck, trunk, and knee flexion angles. Different letters above the bars indicate a significant difference between the angles for each body segment based on a post-hoc test (Wilcoxon signed-rank test).

Gender had a significant effect on postural discomfort (*p* < 0.001). Males (mean ± standard deviation: 4.9 ± 0.1) revealed greater postural discomfort than females (4.0 ± 0.04) (Figure 2). Postural discomfort was significantly affected by the joint angles of individual body segments (all *p*-values < 0.001). A neck angle of 40° showed higher postural discomfort (mean difference: up to 1.0) than the other angles (all *p*-values < 0.001) (Figure 2). A trunk angle of 80° showed greater postural discomfort (mean difference: up to 1.2) than the other angles (all *p*-values < 0.001). A knee angle of 60° exhibited greater postural discomfort (mean difference: up to 2.2) compared to the other angles (*p*-values < 0.001).

**Figure 3:**
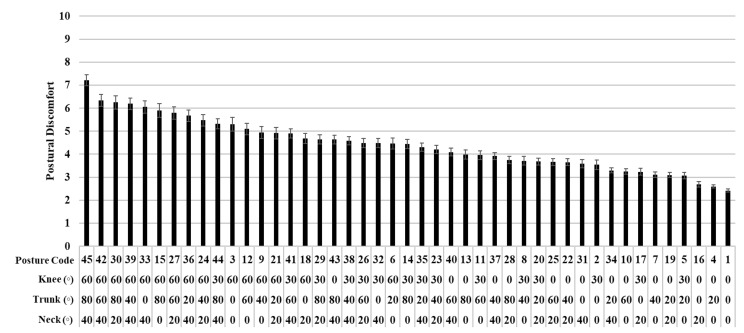
Means and standard errors (error bars) of the postural discomfort by the 45 combined posture codes in terms of the neck, trunk, and knee flexion angles. They are sorted from the largest postural discomfort to the smallest one.

Postural discomfort was significantly affected by the combined postures (*p* < 0.001). Posture code 45 (neck angle: 40°, trunk angle 80°, knee angle 60°) showed the highest postural discomfort (7.2), whereas posture code 1 (zero angles for the neck, trunk, and knee) revealed the lowest discomfort (2.4) (Figure 3).

Three clusters were extracted based on the cluster analysis (Table 3). Although clusters 1 and 2 showed a greater number of observations than cluster 3, no cluster contained an extreme number of observations. All clusters had comparable average and maximum distances from the observations to the centroid of each cluster, which indicated a similar variability of observations within each cluster. For the distance between the cluster centroids, the distance between clusters 1 and 2 was the shortest, whereas the distance between clusters 1 and 3 was the longest. This indicated that cluster 1 had a more similar pattern with cluster 2 than cluster 3.

For the number of observations and the proportion of males and females in each cluster, cluster 3 showed the highest proportion of males but the lowest proportion of females (Table 4). Cluster 1 showed the opposite pattern to cluster 3 by having a larger proportion of females.

**Figure 4:**
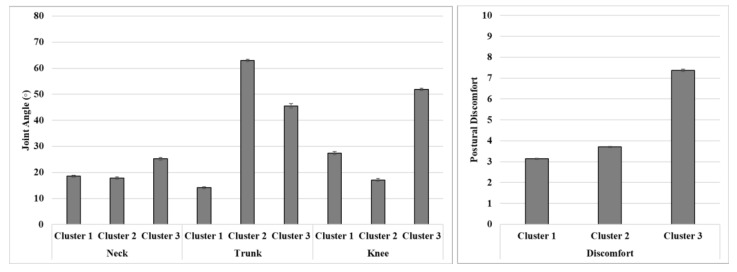
Means and standard errors (error bars) of the neck, trunk, and knee flexion angles, as well as postural discomfort for the three clusters.

There was a significant difference in postural discomfort between clusters (*p* < 0.001). As seen in Figure 4, cluster 1 contained low discomfort (3.1 ± 0.02), low neck (18.5 ± 0.4°) and trunk (14.1 ± 0.3°) angles, and a moderate knee angle (27.4 ± 0.6°). Cluster 2 contained moderate discomfort (3.7 ± 0.03), low neck (17.8 ± 0.4°) and knee (17.1 ± 0.5°) angles, and a high trunk angle (63.0 ± 0.4°). Cluster 3 contained high discomfort (7.4 ± 0.1), a moderate neck (25.2 ± 0.5°) angle, and high knee (51.9 ± 0.4°) and trunk (45.5 ± 0.8°) angles.

## 4. Discussion

This study evaluated the effect of gender, flexion angles of the neck, trunk, knee, and the combined postures on postural discomfort, and identified different postural discomfort patterns using cluster analysis. The aforementioned variables significantly affected postural discomfort. Three clusters were extracted to distinguish the postural discomfort levels. The high discomfort group was male-dominant and tended to have high knee and trunk angles and a moderate neck angle. This was in line with a previous study demonstrating that the more joints that were involved in non-neutral postures, the higher the levels of whole-body postural discomfort that occurred [18]. The low discomfort group was female-dominant and contained low neck and trunk angles and a moderate knee angle.

Gender significantly affected postural discomfort. Males showed greater postural discomfort than females. A majority of the males were assigned in the high and moderate discomfort groups, whereas more females were assigned in low and moderate discomfort groups based on the cluster analysis. This could be partially related to gender differences in flexibility or ranges of motion of joints. It has been known that females tend to have a greater range of motion than males [20]. In order to maintain the combined postures, males could experience greater muscle stiffness than females, which could lead to higher postural discomfort. This suggests that gender-specific postural assessment would be an important factor to consider.

The knee angle was a significant factor that was associated with the degree of postural discomfort. The top five combined postures regarding postural discomfort included a 60° knee angle. In addition, a 60° knee angle also showed higher mean postural discomfort than the other joint angles. This was consistent with a previous study showing that knee flexion was an important factor affecting the discomfort of leg postures [1]. They assessed the postural load of lower limb postures based on perceived discomfort [1]. They mentioned that leg postures were closely related to postural stability and their influence on the whole-body postural load should be fully reflected when evaluating postural discomfort. Sustained contraction of thigh muscles to maintain knee-flexed postures could affect the perception of whole-body discomfort [1]. This indicates that a variety of leg postures and detailed divisions of knee flexion angles could be an important factor to assess whole-body postural stress.

The trunk angle was closely related to postural discomfort, which was supported by a previous study showing that the low back posture was a dominant factor affecting whole-body postural stresses [18]. It is well known that a non-neutral trunk posture is one of the occupational risk factors associated with low back pain [21]. There is a positive association between low back pain and a trunk flexion greater than 20° and the degree of association increases with greater flexion angles of the trunk [21]. This was in line with the present study, which showed that the high discomfort group consisted of mean trunk flexion angles of 45.5°. Increased trunk flexion could lead to a greater moment arm between the center of gravity of the torso and the lumbar spine. This could increase the external moment on the lumbosacral joint, which could lead to a greater muscular tension of erector spinae to stabilize the torso. These effects could stimulate mechanoreceptors located in the muscles and tendons, causing postural discomfort [22].

The neck angle was moderately associated with postural discomfort levels. Although the high discomfort group showed a greater neck angle (25.2°) than the other groups, the low and moderate discomfort groups showed a limited difference in their neck angles (18–19°). This indicated that the neck angle had a relatively lower impact on whole-body discomfort compared to trunk and knee angles. This was consistent with a previous study showing that the perceived discomfort-based ranking of the neck was lower (less discomfort) than the back, hip, and knee joints [12].

Although this study was carefully designed and controlled, there were several limitations. First, subjective discomfort was only considered when assessing postural stress. Self-reporting discomfort values could have limited repeatability regarding outcomes. Although the subjective discomfort collected in this study was closely related to gender and joint angles, directly measuring the muscle activities of a localized body segment would be helpful to assist the findings of this study. Second, only the short-term effect of different static postures was evaluated in this study. Since each participant maintained each posture for 3 s, postural fatigue could not be addressed in this study. Future studies may explore the effect of prolonged static postures on postural discomfort levels. Third, participants were limited to young adults (i.e., university students) in this study. Elderly participants may reveal different levels or patterns of postural discomfort. Different age groups and their effects on postural discomfort could be further studied in the future. Lastly, only static postures were considered in this study. Dynamic motions of the joints and their association with discomfort could be further investigated.

## 5. Conclusions

Gender and flexion angles of the neck, trunk, and knee were important factors that affected whole-body postural discomfort. Males showed greater postural discomfort than females while maintaining a variety of combined postures. Postural discomfort increased as the joint angles increased. Cluster analysis was used to effectively classify 45 combined postures into three different postural discomfort groups, which were closely related to gender and the neck, trunk, and knee angles. The high discomfort group showed male-dominance and a high degree of knee and trunk flexions, whereas the low discomfort group exhibited female-dominance, low trunk flexion, and moderate knee flexions. The neck angle showed a relatively lower impact on postural discomfort compared to the other variables. The findings of this study would be useful for advancing the design of postural assessment tools, especially for unsupported static postures.

## Figures and Tables

**Table 1:** Mean (SD) of participants’ demographic information, including the number of participants, age, height, and weight.

Demographic Variable	Male	Female	Total
Number of participants	42	53	95
Age (years)	24.5 (2.5)	21.0 (2.9)	22.5 (2.8)
Height (cm)	176.5 (7.2)	165.2 (5.9)	170.8 (6.5)
Weight (kg)	69.2 (8.5)	55.1 (6.5)	62.1 (8.1)

**Table 2:** Description of the 45 combined postures in terms of the neck flexion (0°, 20°, and 40°), trunk flexion (0°, 20°, 40°, 60°, and 80°), and knee flexion (0°, 30°, and 60°) angles.

**Posture Code**	**1**	**2**	**3**	**4**	**5**	**6**	**7**	**8**	**9**	**10**
Neck (°)	0	0	0	0	0	0	0	0	0	0
Trunk (°)	0	0	0	20	20	20	40	40	40	60
Knee (°)	0	30	60	0	30	60	0	30	60	0
**Posture Code**	**11**	**12**	**13**	**14**	**15**	**16**	**17**	**18**	**19**	**20**
Neck (°)	0	0	0	0	0	20	20	20	20	20
Trunk (°)	60	60	80	80	80	0	0	0	20	20
Knee (°)	30	60	0	30	60	0	30	60	0	30
**Posture Code**	**21**	**22**	**23**	**24**	**25**	**26**	**27**	**28**	**29**	**30**
Neck (°)	20	20	20	20	20	20	20	20	20	20
Trunk (°)	20	40	40	40	60	60	60	80	80	80
Knee (°)	60	0	30	60	0	30	60	0	30	60
**Posture Code**	**31**	**32**	**33**	**34**	**35**	**36**	**37**	**38**	**39**	**40**
Neck (°)	40	40	40	40	40	40	40	40	40	40
Trunk (°)	0	0	0	20	20	20	40	40	40	60
Knee (°)	0	30	60	0	30	60	0	30	60	0
**Posture Code**	**41**	**42**	**43**	**44**	**45**					
Neck (°)	40	40	40	40	40					
Trunk (°)	60	60	80	80	80					
Knee (°)	30	60	0	30	60					

**Table 3:** Results of the cluster analysis, where the number of observations, average and maximum distance (*σ*) from the centroid of each cluster, and the distance (*σ*) between the cluster centroids are summarized.

**Parameters**	**Cluster 1**	**Cluster 2**	**Cluster 3**
Number of observations	1624	1553	1057
Average distance from centroid	1.8	1.8	1.9
Maximum distance from centroid	3.0	3.3	3.3
**Distance between Cluster Centroids**	**Cluster 1**	**Cluster 2**	**Cluster 3**
Cluster 1	0	1.8	2.5
Cluster 2	1.8	0	2.3
Cluster 3	2.5	2.3	0

**Table 4:** Number of observations and the proportion (%) of males and females in each cluster.

Gender	Variable	Cluster 1	Cluster 2	Cluster 3	Total
Male	Number of observations	545	632	676	1853
	Proportion (%)	29.4	34.1	36.5	100
Female	Number of observations	1079	921	381	2381
	Proportion (%)	45.3	38.7	16.0	100
